# Locating the Social Origins of Mental Illness: The Explanatory Models of Mental Illness Among Clergy from Different Ethnic and Faith Backgrounds

**DOI:** 10.1007/s10943-016-0191-1

**Published:** 2016-02-13

**Authors:** Gerard Leavey, Kate Loewenthal, Michael King

**Affiliations:** Bamford Centre for Mental Health and Wellbeing, Ulster University, Magee Campus, Northlands Road, Derry-Londonderry, BT48 7JL Northern Ireland, UK; Department of Psychology, Royal Holloway, University of London, Egham, UK; Division of Psychiatry, University College London, 6th Floor, Maple House, 149 Tottenham Court Road, London, W1T 7NF UK

**Keywords:** Clergy, Psychiatry, Collaboration, Explanatory models, Causal attributions

## Abstract

Clergy have historically provided ‘healing’ through various spiritual and medical modalities and even in modern, developed welfare economies they may still be an important help-seeking resource. Partnerships between religion and psychiatry are regularly advocated, but there is scant research on clergy explanatory models of illness. This paper aimed to explore their relationship with psychiatry and to examine how clergy in various faith groups conceptualised mental health problems. In this qualitative study using in-depth interviews, these issues were explored with 32 practising clergy in the UK from a range of different Christian, Muslim and Jewish faith organisations and ethnic backgrounds. This paper presents findings related to clergy explanatory models of mental illness and, in particular, how the social factors involved in causation are tinged with spiritual influences and implications, and how the meanings of mental distress assume a social and moral significance in distinctive localised matters.

## Introduction

Much of the healthcare activity is undertaken in the community by non-healthcare individuals and agencies whose beliefs and activities on caring and healing are generally covert (McGuire 1988). Thus, while the complex factors involved in help-seeking behaviour are well documented in the medical sociology literature (Young 2004; Mechanic 1995), less examined in Western settings is the pivotal role of community agencies and individuals such as clergy in help-seeking pathways.

Religion and faith-based organisations are important to health and welfare provision in a number of ways. The ethnic makeup and ideological profile of many Western societies, which might otherwise have expected to move towards homogeneity and secularism (Bruce 2002), globalisation has generated considerable pluralism and a more variegated religious environment. For instance, Pentecostalism and other branches of charismatic Christianity are rapidly expanding among the African diasporas in English and other European cities (Martin 2002; Hunt 2001), as well as in some white communities (Robbins 2004). Pentecostalism is the only branch of Christianity in the UK not in decline (Hunt 2001) and, after Christianity, Islam is the largest religion in the UK (ONS 2003). Religion-motivated violence has provoked intense state surveillance within some minority communities, particularly Muslim populations in Western countries (Juergensmeyer 2003).

More specifically, spiritual and religious beliefs remain integral to the identity of many people influencing behaviour and health in both simple and complex ways. There is evidence that spirituality and religion may be an important coping strategy in mental illness, leading to positive outcomes for sufferers (Pargament 1997; Dillon and Wink 2007). Additionally, public health providers are urged by service user organisations and other commentators to collaborate with faith-based organisation in the provision of care (Friedli 1999, 2000; Koenig et al. 2001; Copsey 1997). Although the relationship between mental health services and religion is rarely examined, it is often characterised as one of mutual hostility or suspicion (Bhugra 1997). However, recent decades have seen some signs of cooperation and synthesis (Loewenthal 2007).

### Explanatory Models

Since the 1970s, research in cultural psychiatry, combined with a new interest in language and symbolic systems, has provided a framework for a meaning-centred medicine in which all illness realities are recognised as fundamentally semantic and that all clinical transactions are, to some degree, interpretive. Thus, the healer or physician sifts through information offered by the sufferer, weighing and interpreting the evidence to advance a ‘reality’ which is then presented to, and negotiated with, the patient (Good and Good 1980: 167). In the absence of ‘objective’ evidence, the clinician ‘makes sense’ of the patient’s narrative, attempting a construction of why has this happened, what or who is responsible and what needs to happen to make things better? Thus, explanatory models of mental illness are thought to play an influential role in the timing and the type of help that individuals and their families are able to negotiate, and how cultural beliefs relate to advice and care when it is provided (Callan and Littlewood 1998; Eisenbruch 1990; Helman 1985; Stein 1986).

All religions attempt an illumination about life, death and human suffering while assisting adherents in their management of the human condition through the use of rituals, mythical narratives and beliefs (Smart 2005), and cultural psychiatry has attempted to illuminate how these belief systems differentially influence help-seeking among ethnocultural groups (Rogler and Cortes 1993). At an individual level, some people construe a relationship between personal or family misfortune and sinfulness or transgression; if illness is understood as punishment requiring spiritual expiation, then medical intervention is less likely to be considered (Sheikh and Furnham 2000; Ying 1990; Meyer 2001). At a more social or structural level, clergy are more likely to be sought in contexts where financial and medical resources are scarce or where clergy are positioned as trusted gatekeepers, particularly among ethnic minority and newly arrived communities. For example, clergy residents in black, Muslim and other minority ethnic communities are key providers of social services and may be more consulted for health matters than their white counterparts (Chaves and Higgins 1992; Hatfield et al. 1996; McCabe and Priebe 2004; Young et al. 2003; Levin 1984). Evidence from the USA suggests that faith-based organisations and their clergy are contacted not just by people in need of counselling, but also by suicidal people and those with psychosis and other disorders (Wang et al. 2003).

However, there is concern that clergy may lack confidence in dealing with mental illness (Weaver 1995). Even where suicide is a real possibility, clergy seem poorly equipped to make an appropriate referral (Wang et al. 2003; Weaver 1995). More damaging perhaps, clergy collusion with powerful culturally informed beliefs in witchcraft and demonic possession have come to light in child abuse cases in the UK (Laming 2003; Garret 2006). However, like the clinician, the priest is guided in this encounter by the sufferer’s narrative which, given the context, may be slanted towards a spiritual interpretation and presentation of the problem. Although explanatory models and help-seeking are socially constructed and mediated, hitherto much of the focus has been on the patient’s beliefs, neglecting the complexity created by social connections and meanings, intersubjectivity and power relationships with other social agents including that of the clergy. This presents a considerable gap in our understanding.

Our interest in clergy and faith-based organisations developed through research on ethnicity and pathways into psychiatric services (King et al. 1994; Cole et al. 1995). The significant religious involvement among the sample suggested the need for dialogue between clergy and mental health services but also the need to explore religious conceptualisations of mental illness and how antagonistic views might be reconciled. Previous papers from the current study provided some understanding about the mixture of mental health pastoral care that faith-based organisations are able to offer and the motivations and challenges in managing mental health (Leavey et al. 2007; Leavey 2008).

## Method

A qualitative design was the most obvious and appropriate approach for developing a rich understanding of how clergy perceive, interpret and respond to the sort of phenomena commonly understood as psychiatric, but may indeed be open to alternative explanations. Thus, in order to explore matters likely to be of considerable complexity, a semi-structured interview was deemed most suitable. Furthermore, the approach adopted involved data collection and analysis as interrelated dialectical activities. This has the advantages of flexibility permitting the development of fresh insights and cumulative theory building (Strauss and Corbin 1990: 67). It also provided direction for further recruitment of participants, identified through faith directories and contacted by letter and then telephone. The interviews were conducted face-to-face at the ministers’ places of worship using a topic guide developed for the study and based on issues that were identified from the literature on religion and mental health. These related to: (a) explanatory models of mental illness; (b) discernment of mental illness; (c) mental health training; (d) aspects of pastoral care and (e) contact and collaboration with mental health services. Each interview lasted between 60 and 180 min with an average duration of 90 min.

### Sample

Thirty-two interviews were completed in a sample that contained 19 Christian ministers, 6 rabbis and 7 imams. The clergy, all men, aged between 37 and 68 years came from a range of religious and ethnic backgrounds. The Christian clergy were English, African, African–Caribbean or South Asian. Where clergy are described as ‘mainstream’ this is simply to indicate the larger, more established churches—the Anglican and Catholic. The imams were from Bangladesh (Bruce 2002), India, Turkey and Kenya. The rabbis were all English-born except one person from South Africa. As indicated previously, most clergy (*n* = 30) worked in London. Although predominantly from deprived inner-city areas, clergy from a range of socio-economic settings were represented. Among the informants, there were three medically trained and qualified doctors, one of whom had been a psychiatrist. Two other clergy had nursing backgrounds in mental health and learning disability. Other clergy had experience of chaplaincy in psychiatric hospitals and training in counselling.

## Analysis

Following professional transcription, each transcript was read several times in combination with audio-reviewing in order to check for any transcribing inaccuracies, to facilitate immersion with the broad views of the individual clergy and to form initial ideas and hypotheses. All interview transcripts were first analysed using the standard systematic processes of qualitative analysis: descriptive and inferential coding, memoing and data display (Miles and Huberman 1994). A more complete description is provided in a previous paper (Leavey et al. 2007).

### Overview

In the study as a whole, the primary interest was the categorisation and elucidation of clergy concepts of mental illness and how these interconnect to wider issues of help-seeking and psychiatric collaboration. Ethnicity, variation in pastoral approaches, literal interpretation of sacred text, culture and secularism evolved as key factors in the analytical framework. Many of the causal attributions offered by the clergy resemble other lay explanations of mental illness (Jorm 2000; Jorm et al. 1997). However, the clergy community concerns revealed how explanatory models of mental illness tend to be underpinned by sensitivity to localised moral and sociopolitical matters. Moreover, mental illness tends not to be seen as simply an individualised, disease entity, but rather as symptomatic of more pervasive spiritual and social malaise. In a companion paper, clergy beliefs focusing solely on the supernatural and mental illness are explored (Leavey 2012), but in this paper it is apparent that clergy views of the social origins of mental illness do not involve a solely ‘secular’ scientific perspective, but a holistic/non-dualistic synthesis of spiritual and social features.

## Causal Attributions

The causal attributions for mental illness were grouped into five main clusters or dimensions. These are as follows: (a) *Biomedical* attributions such as brain chemistry imbalance, brain damage and organic problems related to alcohol and drug use; (b) *Personal life events*, which affect individuals across all social classes (although there may be a social class effect related to incidence, impact and outcome). This category includes causal attributes such as bereavement, relationship problems, work stress and isolation; (c) *Structural*: conditions at a social level and which contains common socio-economic deficits such as poverty, unemployment, poor housing, migration, experience of racism and discrimination; (d) *Modernity,* contains problems that are associated with modern living and is closely related to materialism. This category therefore contains attributes such as secularism and loss of religious identity; (e) *Religious*–*Supernatural*—contains non-natural causes of mental illness such as demonic possession or oppression, witchcraft and engagement with the occult.

Categories that tend towards wider explanations are porous, often not completely demarcated. Thus, clergy commonly suggest close causal links between different categories and attributions. For example, a supernatural explanation of illness may suggest that an individual’s lifestyle, socio-economic circumstances or personal tragedy may leave them vulnerable to supernatural exploitation and demonic exploitation. In other instances, mental illness itself can lead to demonic possession or oppression.

## Biomedical

The ministers rarely discussed the aetiology of mental illness in biomedical terms. Here, the role of biology in the development of mental illness tended to be offered as unicausal, embedded within a genetically determined disability rather than an interplay between nature and environment and often described as a ‘chemical imbalance’ in the body affecting the brain.

I think there are physical causes; there are some kinds of mental illness that you can treat with drugs because it’s about imbalances in enzymes and hormones (A2)

Commonly, mental illness was regarded as a continuum with depression and anxiety at one end and schizophrenia at the other, more serious end. However, one orthodox rabbi (J2) who accurately distinguished between depression and severe mental illness, also offered a clear biomedical explanation for mental illness suggesting chemical and organic causes in addition to shock and trauma. Another rabbi vehemently maintained that most psychiatric problems are socially produced, but nevertheless suggested that there are people who have mental and physical problems as a result of their body’s inability to produce specific vital chemicals.

The only people who need drugs is if for some reason the body can’t produce certain enzymes but however socially close and developed and comfortable and secure they are, the body is just not producing that. Some people, the body doesn’t produce, so there are mental problems, you provide it artificially in some form of drug (J1).

While clergy generally articulated a conventional Western understanding of genetic processes, that is, the transmission and inheritance of disorders, the emphasis on family inheritance described by Pentecostal clergy relates to the inter-family conduit of non-medical entities, negative spiritual energies or contamination. These explanations do not exclude the commonly accepted notion of genetically inherited diseases but rather, one that is entwined with supernatural inheritance.

Sometimes it runs through a family, it sometimes comes straight through a family. It’s like a long chain; if you’re tracing it back you see something like it coming through the family (P4).

If it is in the family, if there is a trace, there is likelihood it may reoccur to another person who is part of the family or the family tree (P1).

This causal attribution by Pentecostal ministers is also discussed elsewhere (Leavey 2012).

### Drugs and Alcohol

While the association between poverty, stress and drug use was commonly alluded to, only a few clergy described actual contact with people with drug and alcohol problems. These tend to be the mainstream Christian (Anglican or Catholic) ministers who are more exposed because their accommodation is attached to the church and that they are more available to the public (Denney et al. 2008). Cultural and religious factors related to drug and alcohol use may also be relevant in addition to the historical ‘sanctuary’ role of the churches, creating role-conflict among such clergy. However, rabbis and imams perceive a growing problem of substance use in their communities, generally associated with growing alienation and loss of normative values. In addition to this, one rabbi (J4) has observed that people from his community were increasingly using ‘recreational’ drugs in order to cope with work-related stress.

Increasing issues around substance-abuse which seem to be creeping into the middle classes as a crutch and also as a life style thing.

Among evangelical and Pentecostal clergy, alcohol and drug use suggests that either a demonic force has gripped a person, or that addiction may lead to vulnerability to demonic attack. For example, one pastor said that mental illness is a method by which demonic spirits can possess the individual, and the gate is often opened by a sinful lifestyle involving alcohol or drugs (see also Leavey 2012).

## Personal Life Events

The effects of personal life events among community members confront clergy across all the faiths. Bereavement and relationship problems create distress in people’s lives and on occasion provoke a crisis of faith. Clergy are then called upon to provide explanations for suffering or to provide advice and support. Personal life events and stressors are sometimes interrelated with other causal attributions. Thus, a growing consumerism impacts work-life balance to the detriment of family life and relationships.

In addition to life problems, various clergy felt that mental illness was often related to personal vulnerability. Thus, the inability to cope with ‘pressure’ and feeling unwanted were considered important factors. Some clergy also discussed how bereavement could jeopardise spiritual and mental health, in part through grief, but also through engaging with forms of spiritualism outside traditional faith structures.

There was also evidence of an idiosyncratic belief: the lunar cycle was raised, albeit tentatively, as a causal possibility by two ministers, one Catholic and one Anglican.

Someone once said to me that people are affected by the phases of the moon and the people who are religiously obsessed will start to be more, I don’t know whether I believe this or not, but people can be affected in this way - they become sort of hyperactive, they start fidgeting in church, and won’t sit still and they will sort of be far more aggressively mobile, I don’t know whether that’s true or not but it does need people to be honest doesn’t it (Ca3).

## Structural Stress

In this section, we attempt to show the way in which clergy perceive mental illness as a production of distinct social and environmental pressures and conditions that impinge on their respective communities. To some extent, these may be interpreted as either a wider political complaint or resulting from particular ideological (possibly theological) positions about the nature, origins and purpose of suffering generally (theodicy). Predominantly, clergy suggest the cumulative effects of social stressors among ‘people leading perfectly sane lives’ as one minister described it, which ‘trigger something that causes them to go beyond what we would call normally acceptable behaviour’.

While several clergy view the lives of people in the deprived parts of the communities as difficult and beset by distressing problems, there was no consensus among the clergy on the contribution of poverty as a determining factor in the provocation of mental illness. To the rabbis, the pressures of modern society tend to impact on the well-being of individuals, but, in contrast to the dominant Christian view, they downplay poverty as an explanatory factor. Moreover, a Catholic priest (Ca2) suggested that social and environmental factors are not causally related but rather, that socio-economic factors influence the way that the illness is presented and how it is managed, both by the community and immediate family. Thus, while the prevalence of mental illness is similar among the wealthy and poor areas, differences relate primarily to differential visibility, toleration, stigma, access to services and coping resources.

One rabbi (J4), who has worked in contrasting affluent and deprived areas in north London and east London, found not less problems but different causes and issues. He suggested that ‘the whole issue is not linear—it’s quite complex’. Affluence, just like poverty, produces its own particular psychological problems.

I imagined that I would see less of a certain type of phenomenon in terms of nervous breakdowns and stress related problems coming here because this was such an affluent community and in fact that’s *not* the case and its led me to believe that its precisely the demands of creating that affluence and sustaining it and the social expectations that go with that, cause a great deal of the problem (J4).

### Ethnicity

Social stress was also discussed by clergy working in deprived inner-city areas as embedded in their concerns for migrant and minority ethnic populations but, significantly, these represent fairly ethnocentric world views in that the issue of ethnicity and psychiatry was raised by black clergy but not at all by white participants—highlighted as social and political anxieties about the impact of racism and discrimination and the possible misdiagnosis of black people by psychiatrists. For instance, one Pentecostal minister, asked about the possible causes of severe mental illness, began by referring to research which highlighted the high rates of schizophrenia among young black men, findings challenged by him as stereotyping, misinterpretation and misdiagnosis:

Behaviour considered schizophrenic in this country is considered normal in our country…They might shout and they might speak in an aggressive manner and half the time, the things that they say, they don’t really mean them but they only say it in the heat of the moment …but because there’s anger inside. We just don’t think that it’s quite right to brand them that way. (P4)

### Social Exclusion

The themes on the impact of racism on mental health are also congruous with those of a young African-born CoE Minister (A6), a trained medical doctor who changed vocation to become a priest. Describing the process of social exclusion, he outlined how young, black, unemployed males lacking in status are likely to develop persecutory ideas. Described by him as caught in a ‘catch-22 situation’, young black men, when denied access to positions of trust, become frustrated and angry which in turn leads to a reinforcement of their rejection and consequent treatment. Cultural stereotypes, about young African–Caribbeans in particular, contribute to the negative social and mental health outcomes in this group.

His paranoia leads to something else and one day he ends up you know, either getting too physical with someone or destructive and he begins to manifest psychotic symptoms - his social problems has led him into psychological imbalance. Racism, discrimination - only recently people have recognised that racial prejudices can lead people to become socially and mentally imbalanced (A6).

Although framed as a ‘black issue’, racialised or political dimensions of mental illness were generally absent among African Pentecostal clergy whose views indicate an apolitical perspective, reinforced by an overarching belief system. Thus, belief in Jesus helps the individual to surmount all such problems (Leavey 2004).

A further illustration of how explanatory models of mental illness emerge from local concerns is given by a Turkish *hoca* or imam (M5) who argued that the high rates of psychiatric disorder, suicide and self-harm in the Turkish–Kurdish community in north-east London can be attributed to the difficulties faced by many in his community, asylum-seekers and others who have waited many years for the Home Office to confirm the right to remain in the UK. Many in his community have not seen their families in Turkey for several years. This view links with the explanations of mental illness provided by Turkish–Kurdish patients in a previous study (Leavey et al. 2007). According to the imam, isolation and loss of hope drive alcohol use as a way of coping. Moreover, governments foolishly ignore the fact that people have a spiritual side which if not considered and nurtured results in many of the social problems, including crime and mental illness that he sees. Similarly, an Indian-born Anglican clergyman whose congregation contains many people from Sri Lanka associates mental illness with migration due to problems of acculturation and exclusion. Thus, language attainment problems and discrimination force qualified professionals in his community to accept low-paid, menial positions, and this creates mental distress.

## Modernity

### Materialism and Young People

The issue of materialism and its perceived impact on mental health appeared to be a matter of paramount concern to the rabbis and imams rather than any of the clergy from the Christian denominations, the focus of which was problems within families and the mental health of children. The Bangladeshi community provides a useful example. Most of the communities served by the imams in London arrived in Britain since the 1970s. Although this community is relatively poor by London standards, they are also relatively rich by Bangladesh standards. From the imams’ perspective, and probably those of the older generation, younger people, second and third generations are increasingly materialistic and envious and, as a result, vulnerable to mental illness.

I think young people are mostly affected at the moment. Teenagers, maybe someone sees his father doesn’t have enough money and his uncle has two or three cars, a big house (M1).

### Children, Parenting and Authority

For one imam, the difficulty for many families is that children are emotionally and culturally torn between the values and expectations of Western modern society and those of the traditional religious culture on issues such as personal choice, sexual behaviour. Western liberal concepts such as the rights of children have less meaning for most traditional families. To the imams, life in a Western society is presented as loss of control *for* the individual and also as a loss of control *over* the individual where authority, to both the older generation and to Islam, is eroded. For the children of Muslim parents, Western individualism offers a dangerous alternative framework or ideology for living. Thus, there is considerable tension within Muslim families about the choices of conformity to either British society or traditional Islamic society.

It’s the in-thing for your child to have a boyfriend - you’ve got to conform but what’s happened as a result of breaking this control down? The child does not fear any more the authority of the parent (M4).

One rabbi (J5), based in a prosperous part of north London, believed that there is a high prevalence of mental health problems among young men, and this is caused by the high expectations placed upon them by their parents. Conversely, another rabbi (J1) argued that mental illness, suicide and other ‘deviance’ is rare in the Jewish community relative to other ethnic groups, partly explained by religious belief, but also the strength of community solidarity. Nevertheless, he is also concerned about the effects of materialism and the pressures this creates for family life.

This rabbi suggests that the issue of low self-esteem or *self*-*destruction*, as he also calls it, is associated with the problems of globalisation, the loss of community and identity. As he perceived it, mass migration of people from rural communities and into large urban centres provokes a loss of identity and belonging; ‘they lose their structure’ and are vulnerable to destructive lifestyles, poor health and criminality. The social upheaval manufactured by migration is part of the larger picture of the chaos inherent in modernity within which the rabbi very definitely locates stress and psychiatric illness. Our reliance on technology distances us from the protective aspects of nature and also weakens our capacity to cope with stress. Thus, in a pre-modern era people were happier and healthier; cars, central heating, artificial light, lack of ‘real exercise’ (both physical and mental) are contributing factors to poor mental health.

The decline of the family was another factor in the increase in mental health problems, and the rabbi referred to an increasing number of single-parent families, which he considered to be detrimental to the well-being of children. Moreover, he viewed the problems of modern living being played out in the home.

The pressure is growing enormously. Parents are fighting - children don’t see their parents, or the parents come home and the father is so fed up so he also screams ….He might go out to the synagogue and talk with his friend because he can’t cope with what’s going on, the child needs the security of the parent (J1).

Parents, he suggested, are often unaware of the deep insecurities that are being created within their children by the lack of attention being paid to them and very often ‘try to compensate the children with money’. Moreover, in his view, social and personal problems were too easily diagnosed as ‘psychiatric’ (medical), and there is a need to treat their ‘true social origins’. In common with the imams, the rabbi believed that much of the origins of social and mental malaise can be traced to modernity and materialism (envy and greed) and the destruction of authority and structures (family life).

### Secularism

One imam who works across several areas in the UK, suggested that

Disbelief in God is the root cause of every problem, whether it is physical, whether mental, whether spiritual, because [Without God] there is no comfort or peace (M3).

He attributed to secularism in Britain, a widespread social deterioration, even the loss of the British Empire, and an increase in existentialist misery. Interestingly, other African-born clergy from different faith groups also made the connection between secularist growth and the decline of British colonial power in Africa. An Indian-born Anglican priest living in England echoed the ‘secularist-madness’ views expressed by the imams, but these were more socially slanted. Thus, he argued that in India God is at the centre of both the community and the individual’s existence; in consequence, people feel intimately and directly connected to a physical and cosmic network—less likely to be isolated and ‘go off the rails’. Importantly too, embedded in their religious beliefs is a powerful fatalism, which can have some harmful effects, but generally, the acceptance of suffering it offers assuages the demands for ‘things’ or sense of failure when they don’t obtain them. In Western societies, rampant individualism produces high expectations, low self-esteem and considerable anger which he suggested was related to mental illness

In the Indian context you keep God in the centre, even as a child. Some of it may be wron*g*. If you scientifically analyse it may sound very stupid. But here (in England) having yourself at the centre and putting God along with all other things in different compartments, your work, your friends, and religion. You operate here different compartments and at quite distance from each of them. And that creates a big problem because they are not supported (A5)

## Discussion

There is a considerable body of explanatory models and help-seeking literature (Kirmayer et al. 1996; Weiss et al. 1992; Weiss 1997; Cauce et al. 2002), but despite the importance of religious-oriented help-seeking and clergy response to mental illness (Wang et al. 2003; Weaver et al. 2003), the subject has received little attention beyond that of the anthropological literature on indigenous and religious healers in developing societies (Teuton et al. 2007; Ensink and Robertson 1999). However, globalisation and rapid expansion of new migrant communities, particularly in Western cities, should predict a profusion of religio-cultural conceptualisations of mental illness. This diversity adds complexity to any attempt by service providers to incorporate faith-based organisations (FBO) as an adjunct to statutory mental health care provision.

Various studies in Western societies have examined lay beliefs about causes and risk factors for mental illness (Jorm et al. 1997), and the findings from the current study suggest that clergy have a similar degree of comprehension as the rest of the lay public (Jorm 2000). It is believed that biomedical or disease models or illness are more common in Western societies while situational models of distress explanation are more prevalent among traditional and minority ethnic communities (Keyes 1985; Patel 1995). However, in a major survey in Australia, social environmental factors were seen as likely causes of depression, consistent with the epidemiological evidence while genetic factors were considered by only half the population. Perhaps more surprisingly, social environmental causes were also given for schizophrenia (Jorm et al. 1997). In UK and Irish studies, stress in the form of family, unemployment and work pressures was the most commonly noted cause of depression followed by bereavement, heredity and childhood adversity (McKeon and Carrick 1991; Sims 1993; Priest et al. 1996). Additionally, a survey among Germans revealed that psychosocial stress was considered to be the biggest risk factor for schizophrenia followed by biological factors, intrapsychic factors, socialisation and the state of society (Angermeyer and Matschinger 1994). In this study, we detected the possibility that some clergy are not prone to the dualistic mind(soul)/body view of humanity. The biomedical model often merges with a world view in which spiritual influences pervade.

### Situational Explanations

In a study of cultural conceptions of depression, Karasz (2004) suggested that causal attributions of mental illness are generally observed as *situational*, that is distress is generally understood within the context of events and circumstances of the sufferer’s life, such as those construed by the imam working in the Turkish–Kurdish community of north-east London (Leavey et al. 2007). In relation to the clergy’s causal beliefs about mental illness, the notion of situationality, a ‘commonsense epidemiology’ is important and useful. Thus, the findings suggest that clergy, generally, favour a social stress model of illness but which does not preclude other natural and supernatural explanations. Conversely, the African Pentecostal leaders tend not to advance direct social–political explanations. This may be explained in a number of ways. Predominantly, events and occurrences in the world have a spiritual basis and meaning in which events are determined by forces, good or bad, outside of the natural, empirical view. Ill health, bad luck and lack of social success are ultimately determined by spiritual forces and belief in the Holy Spirit links the believer to a power transcending all human barriers, conquering social, personal and bodily ailments (Martin 2002; van Dijk 2002).

What do the findings from the current study say about the position of the clergy in society and their beliefs about the origins of human suffering in the form of mental distress? Certainly, no unitary and definitive clergy explanatory model of mental illness emerged from these interview data. A common thread that may be detected across clergy beliefs is that mental illness, whatever the genetic origins and biological manifestations, is a symptom of wider social and moral malaise. As such, mental illness may be seen as a distinctly clergy interest within specific local contexts. Taking classical ethnological enquiries as a theoretical backdrop, Kleinman defined experience ‘as the felt flow of interpersonal communication and engagements. Those lived engagements take place in a local world and experience is thoroughly intersubjective. It involves practices, negotiations, contestations among others with whom we are connected. It is a medium in which collective and subjective processes interfuse’ (Kleinman 1988: 3). Moreover, experience is moral, because ‘it is the medium of engagement in everyday life in which things are at stake and people are deeply engaged stakeholders who have important things to lose, to gain and to preserve’ (Kleinman 1988: 5). Moreover, Kleinman emphasises both the variation and the intensity of ‘things that matter’ across and within local worlds.

Importantly however, and echoing Kleinman’s understanding of experience, lay conceptualisations of mental illness are typically perceived to be generated much more visibly, by social forces rather than biological determinants, even though the genetic connection is generally acknowledged. Thus, also such models appear to be viewed through each clergy’s spiritual or theological lens, they reflect their respective environments, social fabric and behaviour of community members, communal and personal anxieties and the economies and politics of their respective communities. The focus is seldom on the individual but rather, it encompasses a vision and a diagnosis for societal ills. Thus, beyond the tangible reactions to existential problems of loss and injury, mental illness is a symptom of social turbulence.

The clergy are patently stakeholders, and the vision they provide of Western society and its values is generally a pessimistic one. The problems associated with mental illness are closely related to the destruction of traditional values, individualism and the erosion of the family and community, materialism, lifestyle and the loss of authority; in clergy terms, a breakdown of structures that are imbued with and informed by religious values. For instance, the emphasis given by the imams and rabbis on the problems generated by materialism and the various ills associated with modernity which are posed as threats to the mental well-being of younger people suggests a number of things. First, the moral threat and the mental health threat associated with modernism are presented as co-morbidity. More, importantly perhaps, the concern for the mental health of young people opens a window to a more generalised concern about the undermining of a minority culture through assimilation; a seduction away from religious adherence and community. However, it is noteworthy that this concern about contamination was seldom associated with the older generation. It may be that the older generation is perceived to be somehow more protected from these dangers or perhaps, less exposed.

Explanatory models, however, are not just diagnostic in nature, but rather also, cognitive systems, organising and orientating the help-seeker and helper towards a resolution or relief. It may also simply provide an acceptance, fatalistic or stoical. Although often fragmentary and inchoate, explanatory models suggest a strategy or at least some resolution possibilities. We can view the explanatory models of clergy as moral manifestoes in which individual behaviour is important, but deeper structural change is regarded as essential to any amelioration of human misery. It is here that clergy conceive of the religious involvement, articulating the need for change and return to tradition, albeit somewhat rosily depicted. In clergy terms, the secular mindset has removed a spiritual dimension from social activity—the consequences of this deletion have both social and spiritual consequences.

Several questions emerging from the diverse local clergy perspectives seem pertinent. Primarily, how might these models of illness impact on the way that pastoral care is provided? At an individual level, we would suggest that generally clergy approaches to help-seeking will not be very much affected by the world views that they espouse. Thus, other data from this study suggests that clergy, irrespective of faith grouping, will still contact or refer to mental health services. That is not to say that clergy uniformly hold psychiatry in high esteem but rather, clergy tend to recognise their inability to help and they tend to react pragmatically to these encounters. However, the findings suggest that clergy may be somewhat sceptical of the biomedical model of illness and will develop pastoral care approaches and potential collaboration with mental health services, distinctly influenced by collective and systemic, non-medical and moral/religious assumptions. This may not lead to disastrous confrontation with psychiatry; after all, social psychiatry continues to grow in influence and logically, the ramifications for community problems and community rates of mental health problems tend to be expressed more fully and appropriately at the political level.

It may be that, given the depth of structural community problems that clergy indicate as problematic to mental health, they may be amenable to involvement in community development and public health promotion. Nevertheless, as argued by various black clergy, misdiagnosis and perceived mistreatment of black and minority ethnic people is likely to be a corrosive issue which will certainly need to be disentangled and discussed if collaboration and partnerships are to advance. Similarly, for some clergy, the notion of a non-moral approach to guidance and counselling as is perceived by clergy to be the case in secular forms of therapy is considered distinctly unhealthy and remains a block to referral.

Should we expect clergy views to differ very much from those of their congregation or even the general public? Taking this question in a circular fashion, our expectations of clergy knowledge on mental health somewhat rests upon whether or not clergy and their organisations formally acknowledge or perceive this to be an important part of the pastoral role. Currently, this is not formally acknowledged, nor does mental health appear to form an important (or even superficial) element of training for ministry in most, if not all, faith groups in the UK and elsewhere (Weaver 1995; Leavey et al. 2012). Among individual clergy, the extent to which they involve themselves with mental health depends as much upon matters of utility as it does upon resources. In other words, in what ways might dealing with mental health problems further the aims of the church or elevate the standing of the pastor?

In a limited way, this paper is an attempt to highlight and explore issues that are of importance to clergy in their understanding of mental illness and which may potentially be of use in attempting to improve relations between psychiatry and clergy. However, some caution is urged here. It should be acknowledged that the perspectives explored here are somewhat partial and particular; and while this study highlights various key thematic issues, these certainly should not be considered as exhaustive.
